# Genetic polymorphism of *ADAM17* and decreased bilirubin levels are associated with allergic march in the Korean population

**DOI:** 10.1186/s12920-022-01170-7

**Published:** 2022-02-07

**Authors:** Jaemee Jung, Dahyun Hwang

**Affiliations:** 1 Department of Biomedical Laboratory Science, College of Life and Health Sciences, Hoseo University, 20, Hoseo-ro 79 beon-gil, Baebang-eup, Asan, Chungnam 31499 South Korea; 2 The Research Institute for Basic Sciences, Hoseo University, Asan, Chungnam 31499 South Korea

**Keywords:** *ADAM17*, Allergic march, Bilirubin, TNF-α, Single nucleotide polymorphism

## Abstract

**Background:**

The “allergic march” refers to changes in the frequency and intensity of allergic diseases with age. Classically, the allergic march begins with atopic dermatitis in infancy and leads to asthma and rhinitis as it continues. There are many factors that induce the allergic march; however, TNF-α may play an important role in inducing inflammation. Therefore, the therapeutic potential of TNF alpha-targeting agents is being considered for allergic march treatment.

**Methods:**

We performed a correlation study to determine whether genetic polymorphisms of *ADAM17* and clinical serum values between allergic and normal groups affect disease development by using the cohort data of the Korean genome epidemiologic research project. Gene association study was performed using PLINK version 1.07 (http://pngu.mgh.harvard.edu/–purcell/plink) and other statistical analysis was performed using PASW Statistics (version 18.0, SPSS Inc. Chicago, IL, USA).

**Results:**

*ADAM17* (also called TNF-α converting enzyme or TACE) showed a statistically significant association with the allergic march. The 13 and 8 SNPs in *ADAM17* were significantly associated with asthma and allergies, respectively. Among them, on average, SNP of rs6432011 showed the greatest statistical correlation with asthma (*P* = 0.00041, OR = 1.95, 95% CI 1.35–2.82) and allergies (*P* = 0.02918, OR = 1.35, 95% CI 1.03–1.78). The effect of SNPs in *ADAM17* on transcription factor binding was confirmed using RegulomeDB. The six SNPs are located in the genomic expression quantitative trait loci (eQTL) region and can affect transcription factor binding and gene expression. In clinical serum analysis, bilirubin levels were significantly decreased in the allergic group. The multivariate logistic regression analysis revealed that the low-bilirubin groups indicated a 3.22-fold increase in the prevalence of asthma compared with the high-bilirubin group.

**Conclusions:**

The *ADAM17* gene and low bilirubin levels are associated with the allergic march in the Korean population, which can provide new guidelines for managing this disease progression phenomena.

**Supplementary Information:**

The online version contains supplementary material available at 10.1186/s12920-022-01170-7.

## Background

The “allergic march” describes the progression of allergic diseases from atopic dermatitis in infancy to allergic asthma and allergic rhinitis in childhood [1]. Recently, despite significant developments in the pathophysiology of asthma and rhinitis, no comprehensive cure for allergic diseases has yet been developed. Therefore, the early prediction or prevention of the allergic march is very important. Because the allergic march begins in infancy, preliminary indicators could be identified early and genetic approaches have been considered useful. The differences in individual genetic polymorphisms are important factors in determining susceptibility to disease, so recognizing genetic susceptibility to the allergic march can help prevent this disease.

ADAM17 (a disintegrin and metalloprotease 17) is a member of the ADAM protein family and is a bitopic transmembrane protein that spans the lipid bilayer only once. In the human body, ADAM17 is broadly expressed in the lungs, heart, vessels, kidney, brain, testicle, spleen, and muscles [2]. ADAM17 has proteolytic properties that control the shedding of growth factors, cytokines, adhesion molecules, and receptors [3]. This protein is responsible for the release of more than 80 substances from the cell surface, and TNF-α is one of them [4]. The pro-TNF-α is first synthesized as a type II transmembrane protein and then released soluble TNF-α by ADAM17 [5]. The activation of ADAM17 promotes the secretion of soluble TNF-α, which increases inflammatory activity [6]. TNF-α is a representative pro-inflammatory cytokine that plays a critical role in the pathogenesis of inflammatory diseases, such as asthma and allergic inflammation [7, 8]. Therefore, since its discovery, ADAM17 has been considered a major intervention for treating inflammatory diseases and cancer; research into these applications is ongoing [4]. To date, some studies have reported an association between the genetic polymorphism of *ADAM17* and diseases, most of which are related to Parkinson’s disease [9] and vascular diseases [10, 11].

Bilirubin is an end product of heme metabolism and is regarded as potentially cytotoxic and needs to be excreted [12]. It was previously thought that only high serum bilirubin levels may be related to the outbreak of the disease [13, 14]. Recent studies support the idea of low serum bilirubin levels and disease outbreaks. Bilirubin has recently begun to attract attention as a therapeutic agent because elevated bilirubin has been found to exert strong antioxidative and anti-inflammatory activities in the body [15, 16]. The anti-inflammatory activity of bilirubin has been demonstrated in a variety of inflammatory disease models, ranging from chronic to acute inflammation [17]. The protective effect of bilirubin against respiratory disease or injury was already proved in animal model study [18] and some cohort studies [19, 20].

In this study, we conducted a correlation study to determine whether genetic polymorphisms of *ADAM17* and clinical serum values between allergic and normal groups affect disease development by using the cohort data of the Korean genome epidemiologic research project.

## Methods

### Research subjects

The subject data used in this study were obtained from the Korean Association REsource (KARE), which is part of the Korean Dielectric Epidemiology Study [21]. The inclusion criterion for the study subjects were established as previously described [22]. The purpose of this study was to investigate the relationship between genetic variation identified in the KARE study and the allergic march incidence. This analysis included 8,840 subjects (4,822 men and 4,658 women). Among them, 193 and 528 people were diagnosed with asthma and allergies (skin allergies or rhinitis), respectively, and these participants who had at least one disease were selected as the patient group. Total 40 patients were diagnosed with both asthma and allergies (Additional file 1: Fig. S1). A total of 3,228 people who had never been diagnosed with any disease were selected as the control group. Information on the number of study subjects, such as gender ratios, age, and clinical serum levels, are included in Table 1.

**Table 1:** Basic characteristics of KARE subjects

Characteristics	Case–control analysis					
	Asthma			Allergies		
	Normal	Case	*p* value	Normal	Case	*p* value
Number of subjects	3,228	193	**–**	3,228	528	**–**
Gender [men (%)/women (%)]	1,638 (50.7%)/1590 (49.3%)	70 (36.3%)/123 (63.7%)	** < 0.001**	1,638 (50.7%)/1,590 (49.3%)	183 (34.65%)/345 (65.3%)	** < 0.001**
Age (M years ± SD)	51.04 ± 8.80	55.28 ± 8.64	** < 0.001**	51.04 ± 8.80	50.80 ± 8.28	**0.003**
Total bilirubin (mg/dL)	0.60 ± 0.31	0.53 ± 0.22	** < 0.001**	0.60 ± 0.31	0.57 ± 0.29	**0.014**
Total cholesterol (mg/dL)	196.30 ± 35.97	203.63 ± 41.63	**0.018**	196.30 ± 35.97	199.78 ± 35.76	**0.039**

*P*-values lower than the significance level (*P* < 0.05) are indicated in bold

KARE, Korean Association REsource; M, mean value; SD, standard deviation

### Basic characteristics of *ADAM17* and SNPs selection

We used the GeneCards (https://www.genecards.org/) and compartments (https://compartments.jensenlab.org/Search) databases to determine the genomic location and subcellular location of *ADAM17*. SNPs were selected based on KARE genotyping data. DNA samples were isolated from the peripheral blood of the study participants, and Affymetrix Genome-Wide human SNP array 5.0 (Affymetrix, Inc., Santa Clara, CA, USA) was used for genotyping. The SNPs analyzed in this study targeted SNPs present in the *ADAM17* gene transcript site (gene region ± 5,000 bp). The locations of the SNPs in gene were confirmed by referring to the NCBI Human Genome Build 36 (http://www.genome.ucsc.edu/) from 9,546,862 to 9,613,368 bp at chromosome 2. Samples with genotyping accuracies of less than 98%, high heterozygosity (> 30%), and high missing genotype call rates (≥ 4%) were excluded from further testing.

### Linkage disequilibrium of SNPs in *ADAM17*

To determine the rate of genetic recombination using regional plots, we used Haploview version 4.2 (Whitehead Institute for Biomedical Research, Cambridge, MA, USA) and LocusZoom version 1.1 (http://locuszoom.org/), a web-based plotting tool. In addition, we used the Chinese and Japanese population panel of the HapMap database to confirm the recombination rate between SNPs [23].

### Functional assessment of SNPs in *ADAM17*

Both RegulomeDB and GTEx (Genotype-Tissue Expression) Portal databases are used to find out the association between genotype and gene expression. First, we used RegulomeDB to identify potentially functional SNPs (http://www.regulomedb.org/index). This database elucidates how SNPs in the gene region may affect gene or protein expression and contains information on sensitive sites and binding sites of transcription factors through DNase and scores the effects of diseases on the genome of the SNP based on experimental data. The functional grades ranged from 1 to 6, and SNPs showing the strongest evidence of being regulatory (i.e., affecting the binding of transcription factor) were given a score of 1. Next, it was confirmed whether the expression level increased or decreased according to the genotype in the GTExPortal (https://gtexportal.org/home/). GTExPortal is a site that summarizes genetic mutations and gene expression changes in 44 tissues. At this site, the amount of gene expression in the genetic variation was confirmed.

### Statistical analysis between *ADAM17* and allergic march

Statistical analysis of the association between *ADAM17* and asthma and allergies was performed using PLINK version 1.07 (http://pngu.mgh.harvard.edu/–purcell/plink) and PASW Statistics (version 18.0, SPSS Inc. Chicago, IL, USA). The correlation analysis of the genetic variation between the patient and control groups was based on a dominant genetic model using logistic regression analysis. In the regression analysis, age, region, and sex were treated as covariates, and the significance level for the analysis value was 0.05 or less as the standard. Multivariate logistic regression analysis of the genetic variation in rs6432011 of asthma according to the frequency of total bilirubin level was analyzed based on a dominant genetic model.

## Results

### Genome-wide association study (GWAS) with asthma and allergies

**Fig. 1:**
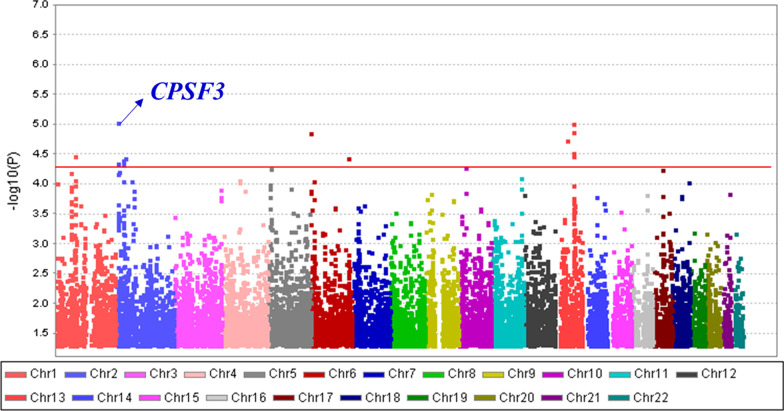
The Manhattan plot for GWAS of asthma in the Korean population. This plot is based on − log(*p*-value) from GWAS analysis against chromosome position, each color represents different chromosome. Red line indicates suggestive significant threshold (*p* = 5 × 10^–5^)

Before proceeding the study, we did GWAS analysis of asthma using the cohort data of the Korean genome epidemiologic research project. The rs12151757 in *CPSF3* showed the highest statistical association (*P* = 9.46 × 10^–6^) with asthma (Fig. 1) and this SNP acts as an eQTL of *ADAM17* (Additional file 1: Table S1). Therefore, we targeted the *ADAM17* and conducted this study. The SNPs in *ADAM17* were used in logistic regression analysis to analyze the correlation between asthma and allergies in patient groups and control groups. *ADAM17* showed a statistically significant correlation (*P* < 0.05) with incidence of the allergic march. The 13 and 8 SNPs in *ADAM17* were significantly associated with asthma and allergies, respectively. The rs6432011 showed the highest correlation with both asthma and allergies. The major allele of rs6432011 is T, and the minor allele is C. The *P-values* of rs6432011 in asthma and allergies were 0.0004 and 0.0291, respectively. In asthma, the odds ratio (OR) of rs6432011 was 1.95, and the 95% confidence interval (CI) was 1.35 to 2.82. For allergies, the OR of rs6432011 was 1.35, and the 95% CI was 1.03 1.78. This suggests that allergic march susceptibility increases when rs6432011 has a minor allele.

### Basic characteristic of *ADAM17* and its SNPs

**Fig. 2:**
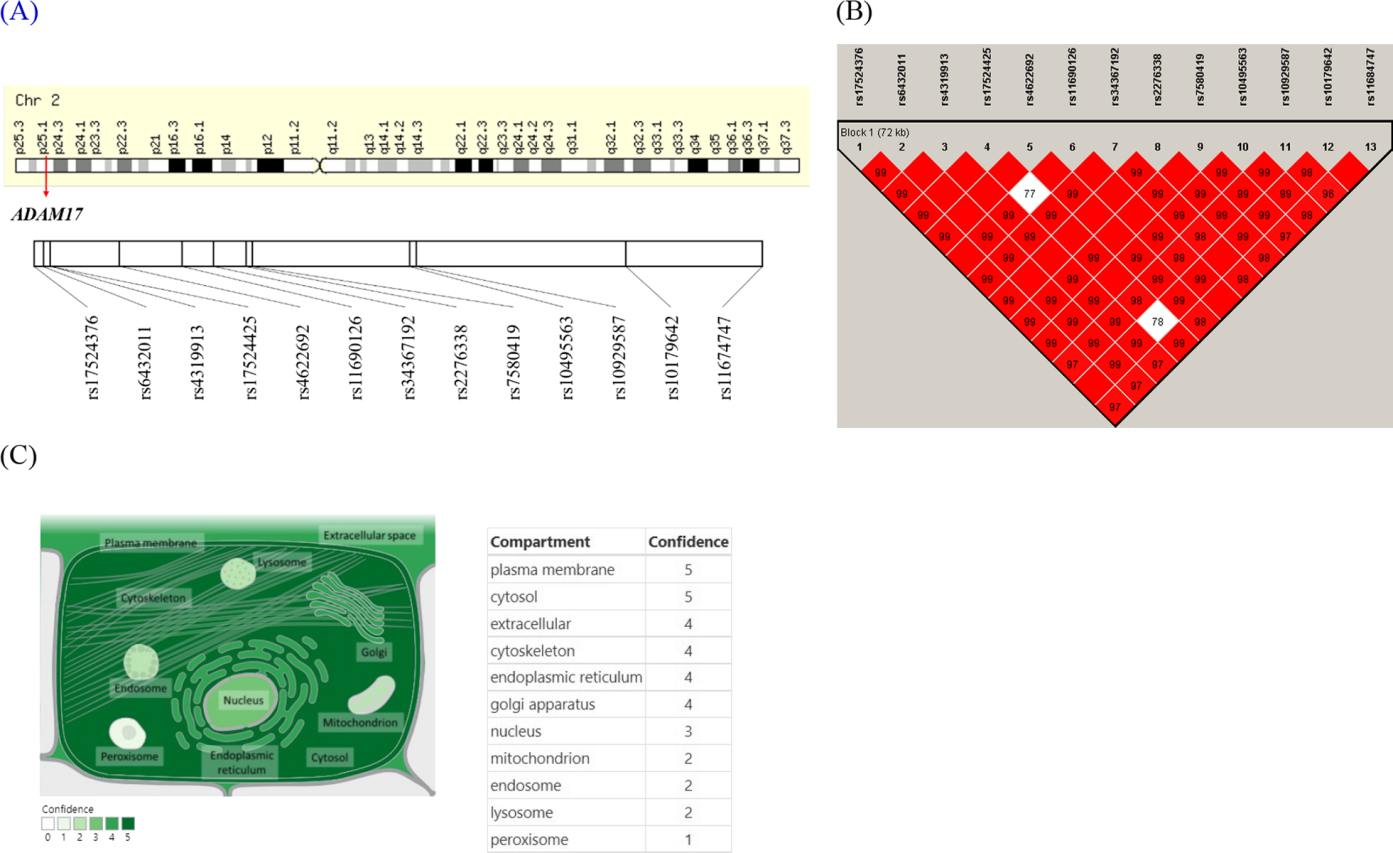
Basic characteristic of *ADAM17*. **A** Gene structure and SNPs location. **B** Linkage disequilibrium (LD) matrix of SNPs in *ADAM17* using Haploview. Red block indicated D’ = 1.0, and the number in the block means the value of D’. **C** Subcellular location and its confidence of *ADAM17* from COMPARTMENTS DB

*ADAM17* is a protein-coding RNA located at 25.1 Chromosome 2. Based on the NCBI human genome build 36, 13 SNPs were identified from the KARE genotype data of the *ADAM17* gene (Fig. 2A and Table 2). All SNPs showed linkage disequilibrium (LD) and moved to one block (Fig. 2B). In subcellular location analysis, *ADAM17* was found to be located in the plasma membrane (Fig. 2C). These results helped us determine the functional location of *ADAM17* in the cells.

**Table 2:** Result of the significant case–control association analysis between SNPs in the *ADAM17* and asthma and allergies in KARE

Gene	Chr	No	SNP	BP	Function	A1	A2	Asthma				Allergies			
								MAF		OR (95% CI)	Additive *p* value	MAF		OR (95% CI)	Additive *p* value
								Case (n = 193)	Control (n = 3,228)			Case (n = 528)	Control (n = 3,228)		
***ADAM17***	2	1	rs17524376	9,542,245	Intronic	A	T	0.042	0.025	1.79 (1.06–3.03)	**0.0293**	0.032	0.024	1.30 (0.88–1.91)	4
		2	rs6432011	9,542,900	Intronic	C	T	0.091	0.050	1.95 (1.35–2.82)	**0.0004**	0.067	0.050	1.35 (1.03–1.78)	**0.0291**
		3	rs4319913	9,543,433	Intronic	T	C	0.091	0.050	1.95 (1.35–2.82)	**0.0004**	0.067	0.050	1.35 (1.03–1.78)	**0.0291**
		4	rs17524425	9,543,455	Intronic	C	T	0.041	0.025	1.76 (1.05–2.95)	**0.0334**	0.031	0.024	1.27 (0.86–1.87)	0.2148
		5	rs4622692	9,550,588	3_UTR	C	A	0.091	0.051	1.92 (1.33–2.77)	**0.0005**	0.067	0.050	1.34 (1.02–1.75)	**0.0353**
		6	rs11690126	9,556,772	Intronic	A	C	0.049	0.025	2.01 (1.23–3.30)	**0.0057**	0.035	0.025	1.42 (0.97–2.06)	0.0673
		7	rs34367192	9,559,929	3_UTR	A	G	0.041	0.025	1.78 (1.05–3.02)	**0.0310**	0.031	0.024	1.26 (0.85–1.86)	0.2341
		8	rs2276338	9,563,240	Intronic	T	C	0.091	0.050	1.94 (1.34–2.81)	**0.0004**	0.067	0.050	1.35 (1.02–1.77)	**0.0314**
		9	rs7580419	9,563,839	3_UTR	A	G	0.091	0.050	1.95 (1.34–2.82)	**0.0004**	0.067	0.050	1.35 (1.02–1.78)	**0.0303**
		10	rs10495563	9,579,661	5_UTR	T	C	0.091	0.050	1.94 (1.34–2.82)	**0.0004**	0.067	0.050	1.35 (1.02–1.78)	**0.0304**
		11	rs10929587	9,580,271	Intronic	A	T	0.089	0.049	1.93 (1.33–2.81)	**0.0005**	0.067	0.048	1.38 (1.05–1.82)	**0.0192**
		12	rs10179642	9,601,147	5_UTR	G	A	0.049	0.025	2.02 (1.23–3.32)	**0.0055**	0.036	0.025	1.46 (1.01–2.13)	**0.0428**
		13	rs11684747	9,614,622	5_UTR	C	T	0.041	0.025	1.78 (1.06–3.00)	**0.0302**	0.031	0.0247	1.27 (0.86–1.87)	0.2164

*P*-values lower than the significance level (*P* < 0.05) are indicated in bold

Chr, chromosome; No, number; BP, base pair; A1, minor allele; A2, major allele; MAF, minor allele frequency; OR, odds ratio; CI, confidence interval. The SNP positions were based on NCBI Build 36 human genome assembly. Age, sex, and residential area were included as covariates in additive genetic models. *P*-values lower than the significance level (*p* < 0.05) are indicated in bold and underlined

### Regional association plot

**Fig. 3:**
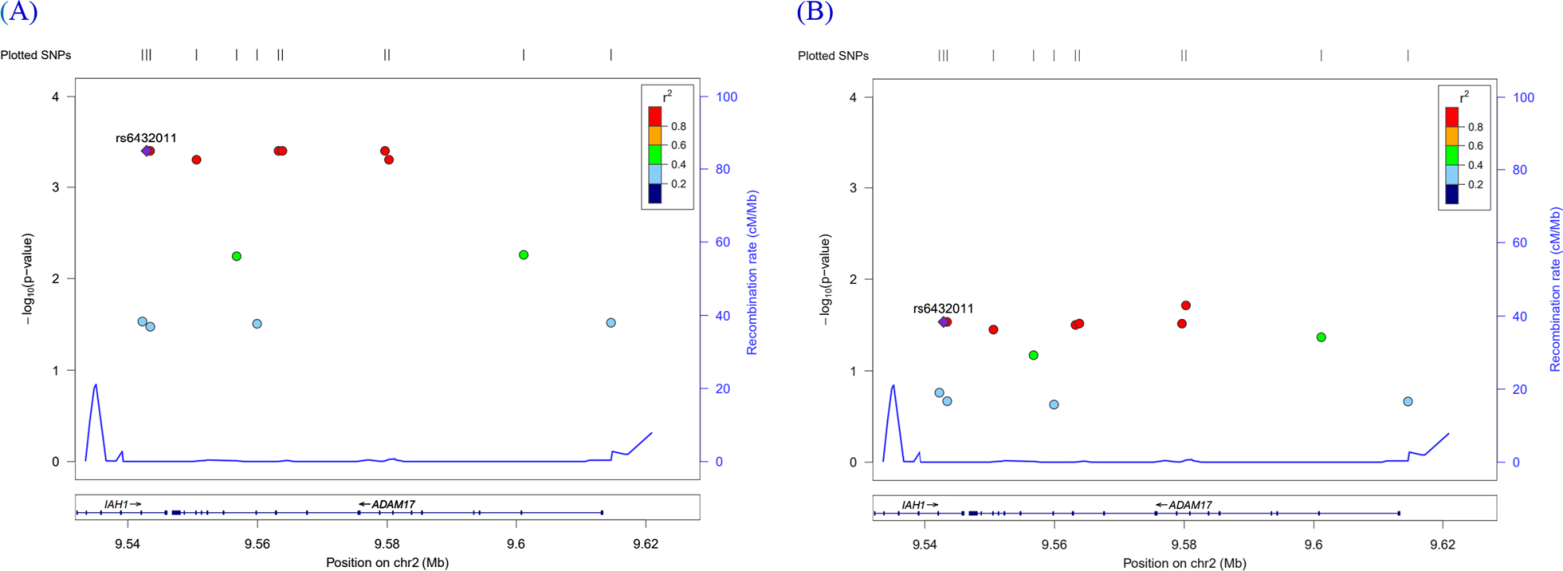
Regional association plots between SNPs in *ADAM17* with asthma (**A**) and allergies (**B**) in KARE subjects. SNP positions in the gene are shown at the top. The Y-axis indicates the statistical significances (− log10 *p*-value) of the association of *ADAM17* with asthma and allergies. The purple diamond (rs6432011) with the most significant SNP number indicates the SNP most strongly associated with the allergic march. SNPs are shown in the indicated colors according to the levels of linkage disequilibrium (*r*^*2*^). At the bottom, the position (Mb) of *ADAM17* on chromosome 2 (NCBI build 36) is shown along with the gene structures

The results of the regional association analysis between SNPs in *ADAM17* and allergic march are shown in Locuszoom version 1.1, a web-based program. Plots of *ADAM17* were generated based on the hg 18 version of JPT + CHB. In *ADAM17*, rs6432011 showed a high significance level in both case groups, which was marked with a purple diamond (Fig. 3). rs6432011 and other statistically significant SNPs were evenly distributed in regions with a high correlation (*r*^2^).

### Effect of SNP in *ADAM17* on gene expression

RegulomeDB was used to determine the correlation of gene expression according to the SNP. As a result, it was confirmed that the SNP with a significant score in the *ADAM17* gene not only affected the binding reaction of transcription factors but also induced a difference in DNase peaks (Table 3). Among them, rs6432011, which had the highest significance level in *ADAM17*, showed a significant score, and it was confirmed that SNP not only affects the transcription factor binding reaction but also has a DNase peak difference. The eQTL, a chromosomal locus that explains variation of the expression trait, is itself *ADAM17* and acts as a motif for OSR1, so it may affect the expression of *ADAM17*.

**Table 3:** RegulomeDB results of SNPs in the *ADAM17*

Gene	SNP	BP	A1	A2	RegulomeDB					
					Score	eQTL	TFBS	DNase	Proteins bound	Motifs
*ADAM17*	rs17524376	9,542,245	A	T	2b	–	+	+	BCL3, POLR2A, RAD21,	NFYB, NFYA, NFYC
	rs6432011	9,542,900	C	T	1f	ADAM17	+	+	–	OSR1
	rs4319913	9,543,433	T	C	1f	ADAM17	+	+	–	–
	rs17524425	9,543,455	C	T	1f	ADAM17, ITGB1BP1	+	+	–	–
	rs4622692	9,550,588	C	A	1f	ADAM17	+	+	–	–
	rs7580419	9,563,839	A	G	1f	ADAM17	+	+	–	–
	rs11684747	9,614,622	C	T	1f	ADAM17, ITGB1BP1	+	+	HDAC1, SMARCA4, KDM5A, RFX3, MBD4, ATF3, ZNF318	–

BP, base pair; A1, minor allele; A2, major allele; eQTL, expression quantitative trait loci; TFBS, transcription factor binding site

At GTExPortal, we checked whether the amount of gene expression increased or decreased when SNP has minor allele. In rs6432011, the amount of gene expression was increased in the case of minor allele (T > C) at muscle and testis tissue (Additional file 1: Fig. S2). An increase in *ADAM17* has been shown to increase the secretion of TNF, causing systemic inflammation and increasing the risk of allergic marches.

### Multivariate logistic regression analysis among genotype, clinical values, and asthma

A comparison of the clinical values between the normal and patient groups is shown in Table 1. A total of 20 clinical values were compared between groups, and only two values (total bilirubin and total cholesterol) showed a statistically significant difference (Additional file 1: Table S2). The total bilirubin level decreased by approximately 11.6% and 5% in the asthma and allergy groups, respectively, compared to the normal group. Total cholesterol slightly increased by approximately 3.6% and 1.5% in the asthma and allergy groups compared to the normal group, respectively.

**Fig. 4:**
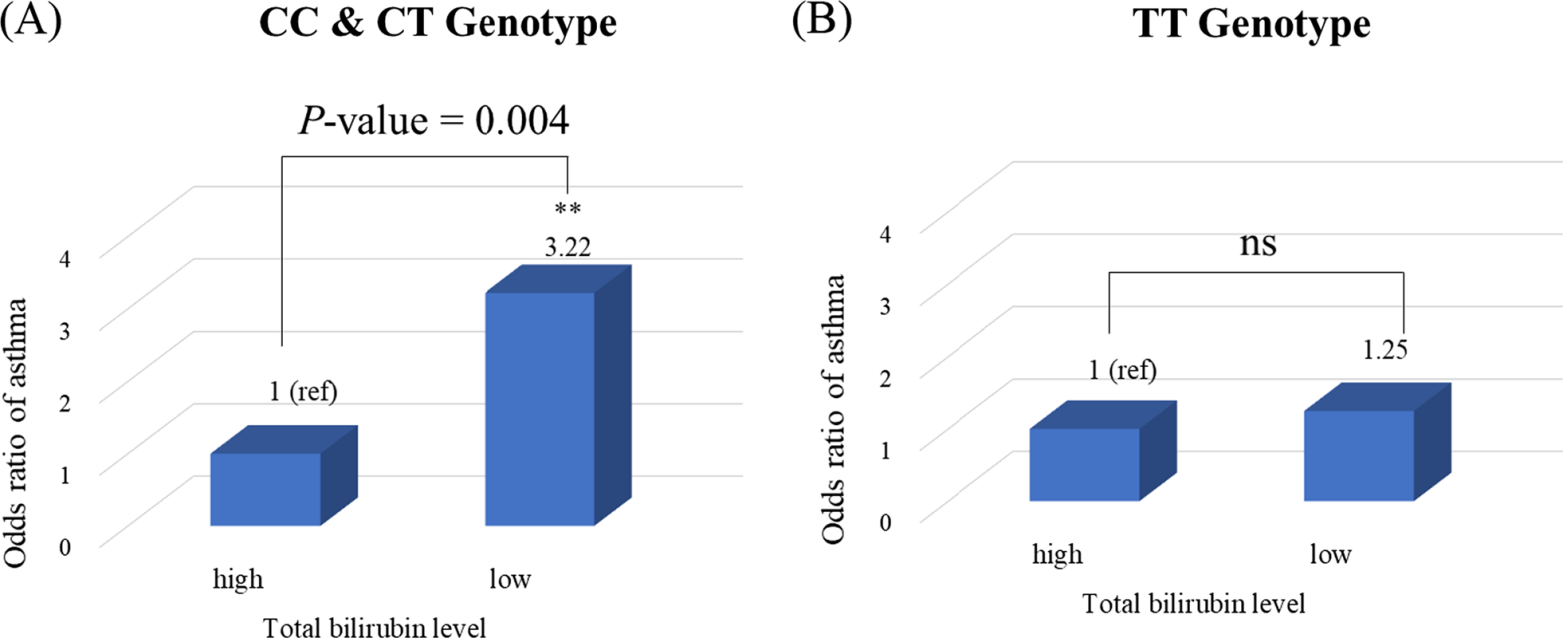
Asthma risk according to total bilirubin levels in CC & CT genotypes and the TT genotype. Multivariate logistic regression analysis was evaluated using rs6432011 data. **A** and **B** show the risk of asthma with decreasing total bilirubin levels for each genotype. Bilirubin levels were divided into high and low level groups based on a threshold of − 0.28 mg/dL. The number at the top of the bar represents the odds ratio value for asthma. The significance (*P*-value) of difference was determined with the Student’s *t-*test. ns, not significant

A multivariate logistic regression analysis was performed on rs6432011 for the OR of asthma, considering the total bilirubin levels and genotypes. The analytical model was based on the dominant genetic model for the minor allele (CC, CT, and TT). The log transformation was used to transform skewed datasets to achieve near-normal distribution on values of bilirubin. The reference point of total bilirubin was divided based on the average value of the control group (− 0.28 mg/dL) (Additional file 1: Fig. S3). Thus, individuals with the TT genotype showed no significant difference in the OR of asthma according to bilirubin levels. However, in the low-level (under − 0.28 mg/dL) CC and CT genotype groups, the OR value of asthma increased about 3.22-fold (95% CI 1.46–7.12) compared to the high-level group. That is, there was no significant correlation when only major alleles were present, but when minor alleles were present, a lower bilirubin level is associated with a higher risk of asthma (Fig. 4).

## Discussion

In this study, we examined whether genetic polymorphisms and clinical values are correlated with incidence of the allergic march. The ADAM17 is involved in TNF-α production, and the association between *ADAM17* and asthma has recently been demonstrated through animal experiments [24]. Therefore, we selected *ADAM17* as a target gene.

According to our findings, the functional position of *ADAM17* in the cell was identified; the highest confidence of subcellular location was in the plasma membrane. *ADAM17* is located in the plasma membrane and acts as the primary enzyme responsible for catalyzing the release of membrane-anchored proteins from the cell surface in metazoan organisms [25]. This process is called ectodomain shedding, and *ADAM17* is responsible for the protease-induced release of more than 70 membrane-linked cytokines, growth factors, and cell surface receptors [26]. In particular, it stimulates the release of TNF-α, causing systemic inflammation and accelerating the allergic march [27]. TNF-α is an important inflammatory agent in the pathological development of allergic diseases, so the potential for treatment with TNF-α targeting agents has recently been considered to treat allergic marches [28]. In this study, the association between *ADAM17* and allergic march was investigated, and the SNPs belonging to *ADAM17* were found to increase the incidence of the allergic march if it had a minor allele. To determine the cause, the correlation between SNPs and gene expression was identified, and six SNPs among the 13 SNPs were located in the eQTL regions. The additional eQTL analysis was performed using GTExPortal. In rs6432011, among 12 tissue data provided, only muscle and testis showed increased *ADAM17* expression with the presence of a minor allele. Moreover, since this database provides data for tissues that are statistically significant only, there is a limitation in that the analyzed data of lung is unclear. Therefore, further experimental complementary studies are needed.

Studies on the allergic march and specific genotypes have been conducted, but few studies have been conducted on the effects of interactions between serum clinical values and *ADAM17* genotype regarding the allergic march. We investigated the correlation of total bilirubin levels between the rs6432011 genotype and the risk of asthma. The C genotype (minor allele) appears to act as a risk factor for asthma, and the interaction between genotype and low bilirubin levels significantly increases the risk of developing asthma. Bilirubin is a yellow pigment produced when red blood cells break down. Lower than normal bilirubin levels are not typically considered concerning because there is no clear link between low bilirubin levels and any medical conditions. However, recent medical correlations between decreased bilirubin levels (hypobilirubinemia) and diseases have been published. Some studies suggest that bilirubin acts as an antioxidant, so if decreased bilirubin levels cause reduced antioxidant effects, there may be a higher risk for various health problems such as coronary artery disease [29], ulcerative colitis [30] and brain lesions [12]. However, there is limited evidence regarding the effect of bilirubin levels on allergic diseases. For this reason, our finding that reduced bilirubin increases the incidence of asthma is novel and elucidates scope for further research. Total cholesterol levels have also been found to exhibit significant differences in the present study, and some studies have examined the correlation between cholesterol and allergies [31, 32]. However, there remains controversy regarding the relationship between allergies and serum cholesterol levels, so we did not perform further analysis.

## Conclusions

The *ADAM17* and low bilirubin levels could be associated with incidence of the allergic march in the Korean population. In particular, groups with minor alleles showed greater sensitivity to allergic diseases, as identified by evaluating total bilirubin levels. Therefore, in this group, managing bilirubin is important for preventing allergic diseases. Although further experimental study will be needed to prove our results, the genetic polymorphism of *ADAM17* and serum bilirubin level might be used as a potential marker for estimating the risk of allergic diseases and can provide new guidelines for the management of the allergic march.

## Supplementary Information

**Additional file 1.** **Figure S1.** Overlapping subject information for asthma (n = 193) and allergy (n = 528). **Figure S2.** Genotype-basedmRNA expression in tissue from the GTEx portal of rs6432011. **Figure S3.** The distribution of total bilirubin. **Table S1.** Results of the Regulome DB of SNPs in the CPSF3. **Table S2.** Results of clinical values between the asthma group and the normal group.

## Data Availability

The information of KARE samples are available by sending a request to the bioresources from National Biobank of Korea, the Korea Disease Control and Prevention Agency, Republic of Korea.

## Abbreviations

eQTL: Expression quantitative trait loci
ADAM17: A disintegrin and metalloprotease 17
KARE: Korean Association Resource
GWAS: Genome-wide association study
LD: Linkage disequilibrium
OR: Odds ratio
CI: Confidence interval
