# Supplementary material for: Genetic polymorphism of ADAM17 and decreased bilirubin levels are associated with allergic march in the Korean population
Source: BMC Med Genomics. 2022 Feb 7;15:21. doi: 10.1186/s12920-022-01170-7 (PMC8822644; doi:10.1186/s12920-022-01170-7)
Supplement: Supplementary file 1 — Additional file 1. Figure S1. Overlapping subject information for asthma (n = 193) and allergy (n = 528). Figure S2. Genotype-basedmRNA expression in tissue from the GTEx portal of rs6432011. Figure S3. The distribution of total bilirubin. Table S1. Results of the Regulome DB of SNPs in the CPSF3. Table S2. Results of clinical values between the asthma group and the normal group. [file 12920_2022_1170_MOESM1_ESM.docx]

**
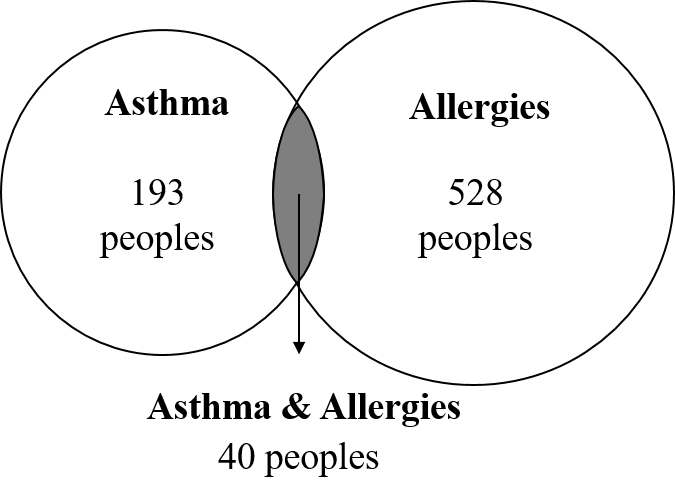
**

**Figure S1**. Overlapping subject information for asthma (n=193) and allergy (n=528). Total 40 patients were diagnosed with both asthma and allergies.


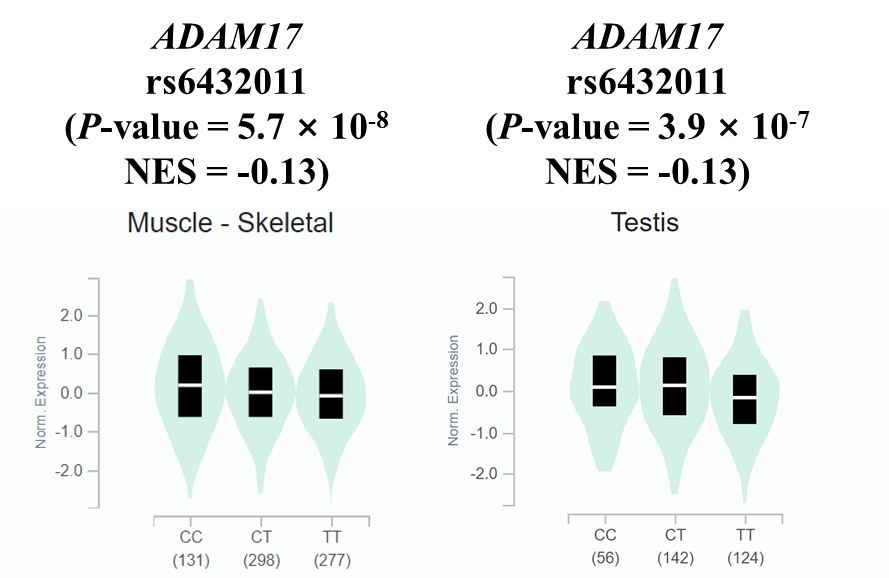


**Figure S2**. Genotype-based mRNA expression in tissue from the GTEx portal of rs6432011. The gene expression level was increased when having a minor allele (C). Significant association between genotypes and gene expression levels in the GTEx portal was detected with the linear regression model and based on a *p*-value threshold determined by a web-based eQTL calculator on the GTEx portal. The white line in the box plot (black) shows the median value of the gene expression at each genotype. *P*-value are calculated with the linear regression model. NES (normalized effect size) means the slope of the linear regression model.

**
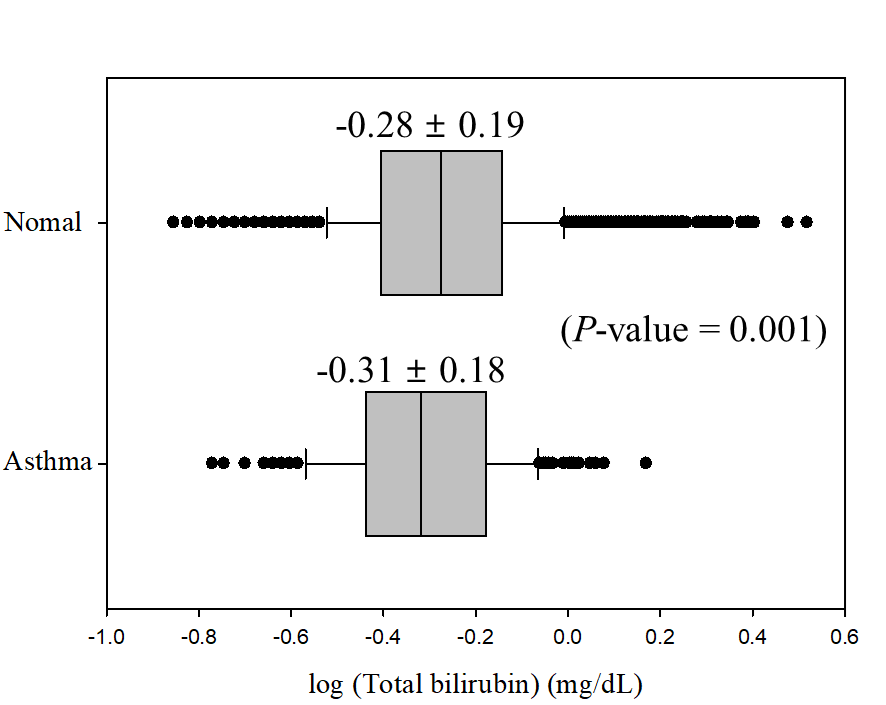
**

**Figure S3**. The distribution of total bilirubin. The log transformation was used to transform skewed datasets to achieve near-normal distribution.

**Table S1**. Results of the Regulome DB of SNPs in the *CPSF3*

| Gene | SNP | BP | A1 | A2 | Regulome DB | | | | | |
| --- | --- | --- | --- | --- | --- | --- | --- | --- | --- | --- |
|  |  |  |  |  | Score | eQTL | TFBS | DNase | Proteins bound | Motifs |
| *CPSF3* | rs11895982 | 9484791 | T | A | 1f | ADAM17 | + | + | NFE2 | NFATC1 |
|  | rs10209595 | 9491117 | C | T | 1f | ITGB1BP1, ADAM17 | + | + | - | - |
|  | rs12464664 | 9491993 | G | A | 1f | ADAM17 | + | + | RFX1 | IRF1, IRF3, IRF7, IRF8, PRDM1, STAT1, TRIM63 |
|  | rs12151757 | 9512596 | C | T | 1f | ADAM17 | + | + | - | - |

Abbreviations: SNP, single nucleotide polymorphism; BP, base pair; A1, minor allele; A2, major allele; eQTL, expression qunatitative trait loci; TFBS, Transcription factor binding site.

**Table S2.**Results of clinical values between the asthma group and the normal group

| Case-control analysis | | | | | | | |
| --- | --- | --- | --- | --- | --- | --- | --- |
| Characteristics | **clinical values** | | | **Characteristics** | **clinical values** | | |
|  | **Normal** | **Asthma** | ***P*-value** |  | **Normal** | **Asthma** | ***P*-value** |
| Glucose  (mg/dL) | 90.78 ± 17.84 | 89.62 ± 13.97 | 0.384 | TCHL (mg/dL) | 196.30 ± 35.97 | 203.63 ± 41.62 | 0.018 |
| Albumin  (g/dL) | 4.50 ± 0.28 | 4.47 ± 0.28 | 0.086 | HDL  (mg/dL) | 49.85 ± 11.84 | 49.23 ± 12.20 | 0.476 |
| BUN  (mg/dL) | 13.71 ± 3.73 | 13.97 ± 4.36 | 0.420 | TRIGLY (mg/dL) | 149.94 ± 110.31 | 163.30 ± 103.06 | 0.102 |
| Creatinine (mg/dL) | 0.82 ± 0.32 | 0.81 ± 0.33 | 0.638 | NA  (mmol/L) | 142.58 ± 2.19 | 142.62 ± 2.10 | 0.840 |
| Calcium  (mg/dL) | 9.61 ± 0.47 | 9.58 ± 0.45 | 0.386 | K  (mmol/L) | 4.51 ± 0.40 | 4.48 ± 0.41 | 0.332 |
| Total Bilirubin (mg/dL) | 0.60 ± 0.31 | 0.52 ± 0.22 | <0.001 | CL  (mmol/L) | 103.05 ± 2.30 | 102.96 ± 2.35 | 0.615 |
| AST  (IU/L) | 25.90 ± 19.53 | 25.73 ± 26.70 | 0.907 | WBC (Thous/μL) | 6.56 ± 1.80 | 6.79 ± 2.01 | 0.082 |
| ALT  (IU/L) | 23.97 ± 29.54 | 23.58 ± 29.03 | 0.856 | RBC (Mil/μL) | 4.42 ± 0.46 | 4.38 ± 0.44 | 0.211 |
| γ -GTP  (IU/L) | 39.69 ± 78.84 | 32.91 ± 44.94 | 0.237 | HB  (g/dL) | 13.61 ± 1.61 | 13.45 ± 1.44 | 0.179 |
| CRP  (mg/dL) | 0.21 ± 0.42 | 0.31 ± 0.52 | 0.013 | HCT  (%) | 41.08 ± 4.66 | 40.90 ± 4.42 | 0.608 |

Abbreviations: AST, aspartate aminotransferase; ALT, alanine aminotransferase; γ-GTP, gamma-glutamyl transpeptidase; CRP, C-reactive protein; TCHL, Total Cholesterol; HDL, high density lipoprotein; TRIGLY, Triglyceride; NA, sodium; K, potassium; CL, chloride; WBC, white blood cell; RBC, red blood cell; HB, hemoglobin; HCT, hematocrit.
